# A rightward saccade to an unexpected stimulus as a marker for lateralised visuospatial attention

**DOI:** 10.1038/s41598-018-25890-y

**Published:** 2018-05-15

**Authors:** Masafumi Sanefuji, Hiroshi Yamashita, Michiko Torio, Daisuke Katsuki, Satoshi Akamine, Yoshito Ishizaki, Junji Kishimoto, Yasunari Sakai, Hidetoshi Takada, Keiko Yoshida, Shouichi Ohga

**Affiliations:** 1 Research Center for Environment and Developmental Medical Sciences, Kyushu University, 3-1-1 Maidashi, Higashi-ku, Fukuoka 812-8582 Japan; 2 Department of Pediatrics, Graduate School of Medical Sciences, Kyushu University, 3-1-1 Maidashi, Higashi-ku, Fukuoka 812-8582 Japan; 3 Department of Child Psychiatry, Graduate School of Medical Sciences, Kyushu University, 3-1-1 Maidashi, Higashi-ku, Fukuoka 812-8582 Japan; 4 Department of Research and Development of Next Generation Medicine, Faculty of Medical Sciences, Kyushu University, 3-1-1 Maidashi, Higashi-ku, Fukuoka 812-8582 Japan; 5 Mental Clinic, Airisu, 3-4-28 Sakurazaka, Chuo-ku, Fukuoka 810-0024 Japan

## Abstract

The human brain is lateralised to the right for visuospatial attention, particularly when reorienting attention to unexpected stimuli. However, the developmental characteristics of lateralisation remain unclear. To address this question, we devised a saccade task applicable for both adults and children. To assess the utility of this system, we investigated the correlation between line bisection test performance and the saccade task for 54 healthy adult volunteers. Participants followed a visual target that jumped 10 times, alternating between two fixed positions across the midline with a constant pace. In both the rightward and leftward directions, saccadic reaction time (RT) to the target jump decreased and reached a plateau from the first to the tenth jumps. Furthermore, we obtained the time required for reorienting in the contralateral hemisphere using the corrected value of the first RT. We found that longer corrected RTs in the rightward saccade were associated with greater deviation to the left in the line bisection task. This correlation was not observed for leftward saccades. Thus, corrected RTs in rightward saccades reflected the strength of individual hemispheric lateralisation. In conclusion, the rightward saccade task provides a suitable marker for lateralised visuospatial attention, and for investigating the development of lateralisation.

## Introduction

Right hemispheric lateralisation in visuospatial attention has long been studied in both pathological and healthy conditions. Impairments in the detection of spatial stimuli in the contralesional hemifield, termed “hemispatial neglect”, is more common and severe following right compared with left hemisphere damage^1–3^. Functional neuroimaging studies revealed that neurologically intact individuals exhibit right lateralised activation of the ventral attention network during stimulus detection^4^. This lateralisation has been found to be particularly prominent when patients or healthy individuals reorient their attention to a stimulus that is unexpected and outside the focus of processing^2,5^. However, there is a high degree of individual variation in lateralisation^6,7^ when quantified with the line bisection test^8^. In healthy adults, line bisection is reported to be slightly biased towards the left, at a group level^9^. This leftward deviation has been interpreted as an overestimation of the left portion of the line, indicating right hemispheric dominance^10,11^. However, because the test is too difficult for young children to accomplish accurately, the developmental characteristics of this form of lateralisation remain unclear.

Saccades are rapid eye movements, and are easily detectable using an eye-tracking system in young children, including infants. To investigate the lateralisation of visuospatial attention, several studies have examined asymmetry in response times (RTs) of reflexive saccades among healthy adults. In these studies, subjects made repetitive saccades to a stimulus that appears randomly at either of two fixed positions in the right and left visual fields. RTs of the saccades are then compared between the right and left visual fields. Based on right hemispheric dominance, detection of the target would be expected to be faster in the left than the right visual field, meaning that RTs would be shorter for leftward saccades in general. However, several studies have not observed asymmetrical RTs^12–14^.

These incongruent results may be related to task designs, which typically involve hundreds of repeated trials. In these tasks, the position and timing of the target are initially unexpected, and thus elicit a reorienting process. Through the repetition of trials, however, subjects may learn the pattern of target presentation. This learning and practice effect may lead subjects to expect the target to appear in either of the fixed positions, potentially lessening the activity of the right-lateralised ventral network that subserves reorientation to unexpected stimuli^2,15^. Furthermore, long periods of repetitive trials could induce fatigue or low alertness, potentially diminishing the leftward shifts in attentional bias (time on task effect)^16–18^ and are too demanding for young children. We devised a square wave saccade task to behaviourally examine the development of the lateralisation of visuospatial attention, with a system specifically designed to be suitable for use with children. The task requires only 10 trials, and takes full advantage of the first trial, which is completely unexpected, and elicits the reorienting process most efficiently. A head-unrestrained eyetracker is used to measure saccadic RT and pupil size, reflecting the degree of unexpectedness. In addition, to assess the utility of this task, we examined its relationship with the line bisection test in neurologically intact adults.

## Methods

### Participants

The sample size was determined based on the following findings. When the significance level and *r*-value were set at 0.05 and 0.5, respectively, the required sample size was 16 subjects or more, according to the table of critical values for the Pearson’s correlation coefficient. Actually, in a study that examined the correlation between bisection performance and lateralisation of RT for a target presented in either the left or right hemifield, Thiebaut de Schotten *et al*. recruited 20 subjects and found a significant correlation (*r* = 0.495, *p* < 0.05; Fig. 2d in their paper)^6^. Thus, we set the desired sample size at 20 subjects for one task. As we expected a considerable number of subjects to be excluded, which was indeed the case, we recruited a total of 54 right-handed healthy volunteers (32 females, aged 20–56 years) for two tasks (see 2.3.2.), with no reimbursement. All participants had normal or corrected-to-normal vision and no hearing difficulty. Their handedness was confirmed using the Edinburgh Handedness Inventory^19^. All participants were naïve to the task and the purpose of the experiment. The present study was approved by the Ethics Committee of the Graduate School of Medical Sciences, Kyushu University (#26–226). Written informed consent was obtained from all participants. All methods were performed in accordance with the relevant guidelines and regulations.

### Instruments

Eye movements were monitored using a remote infrared eye-tracking system (X120; Tobii, Sweden), which does not require head-mounted equipment, chin/head rests, or stickers placed on the forehead. During measurement, the subject is able to move their head freely, and remains trackable as long as the eyes are within a 3D virtual “box” at the correct distance from the system^20^. This system tracks both the eyes with a temporal resolution of 120 Hz and a spatial resolution of 0.5°. Visual and auditory stimuli were presented using a flat-screen display (E2311Hf; Dell, Japan) with a viewable screen size of 508 × 286 mm and a speaker (MM-SPSBA2; Sanwa Supply, Japan). The eyetracker was mounted below the presentation display (Supplementary Fig. S1). The positions of the display and eyetracker were adjustable to the level of each participant’s eyes using a special arm. Tobii Studio® software controlled calibration, stimuli presentation and recordings on a laptop computer (Precision M6800 Mobile Workstation; Dell, Japan). A web camera (HD Pro Webcam C920t; Logicool, Japan) was connected to the laptop for monitoring the general behaviour of the participants.

### Experimental procedures

#### Line bisection test

A black line 20 cm long and 1-mm thick was centred on a horizontal white A4 sheet, with its centre aligned to the participant’s midline^6^. Participants were instructed to mark the subjective centre of the line with a pencil. Each performed 10 trials in total, five with the right hand and five with the left hand. The deviation from the objective centre was measured, and the average performance with both hands was used as the measure of individual deviation. Rightward deviations from the objective centre were coded as positive values while leftward deviations were coded as negative values.

#### Rightward and leftward saccade tasks

**Figure 1:**
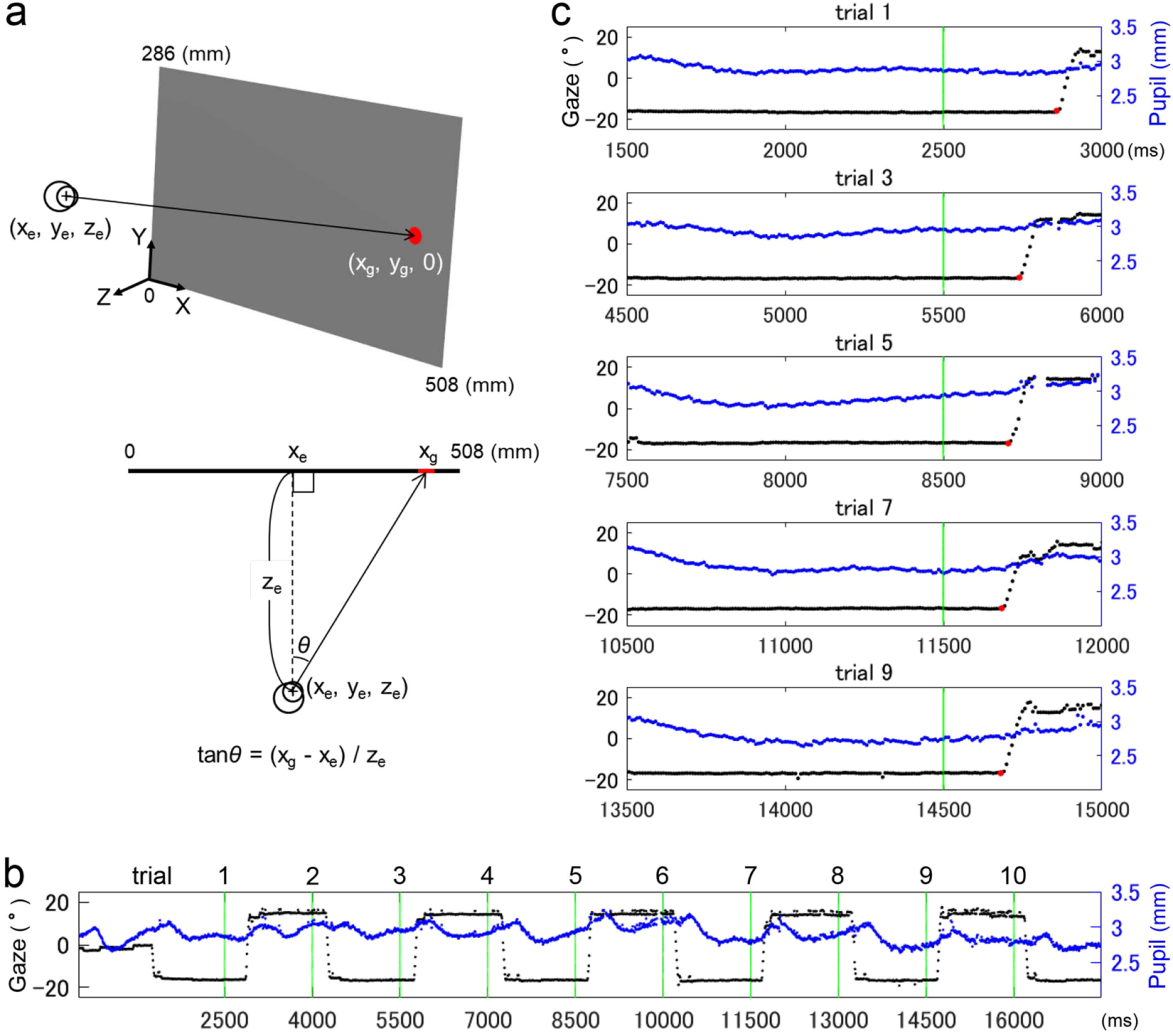
Saccade task. (**a**) Task presentation and calculation of gaze position. The target is now located in the right position on the grey background. Horizontal angles of gaze position (*θ*) were calculated from the coordinates of gaze position on the screen (x_g_, y_g_, 0) and eye position in space (x_e_, y_e_, z_e_). (**b)** A whole trace of gaze position (black) and pupil size (blue) for a representative participant in rightward task. Green vertical lines represent the onsets of target jump. (**c)** The extracted traces for the odd trials. The traces are presented 1000 ms before and 500 ms after the jump onsets. Red dots represent the initiation of saccades.

Participants were seated in front of the centre of the screen at a distance of approximately 75 cm. Participants gaze was calibrated using the “Regular” calibration method provided by the manufacturer (https://www.tobiipro.com/siteassets/tobii-pro/user-manuals/tobii-pro-studio-user-manual.pdf). Briefly, a dot was presented successively in five different positions on the screen, and participants were instructed to fixate on it. Participants performed a speech task and the saccade task, and the latter was analysed in this study. Participants were instructed only to follow a target. The target was a red-filled circle 21 mm in diameter (approximately 1.6° of visual angle) on a grey background. The luminance levels of the circle and the background were 40 cd/m^2^. After 1.0 sec background presentation, the target appeared then jumped 10 times alternately between the two horizontal positions located 200 mm (around 15°) to the left and right from the centre of the screen (Fig. 1a). In the rightward task, the target emerged first in the left position, then jumped to the right position, producing the first saccade towards the right. In the leftward task, the sides were horizontally flipped, generating the first saccade towards the left. The jumps had relatively large visual angles (around 30°), which would be expected to enhance behavioural asymmetries of attention^21^. Each target lasted 1,500 ms, corresponding to a pacing frequency of 0.33 Hz. This frequency is known to elicit reactive saccades, typically with RTs over 100 ms, whereas higher frequencies (e.g., 0.9 Hz) elicit other processes of procedural learning^22^, encouraging predictive saccades, often with negative RTs^23–25^. To maintain vigilance among participants, a tone (1000 Hz, 65 dB, 500 ms) was presented in synchrony with each onset of the target. There were no temporal gaps or overlaps between the offset of the target and the onset of the next target. The task finished in 17.5 sec. Half of the participants performed a rightward saccade task and the other half performed a leftward saccade task.

### Analyses of saccade task

#### Calculation of saccadic RT and pupil dilation

All data processing was performed offline under MATLAB (MathWorks, USA). The eyetracker provided the coordinates of gaze point on the screen and eye location in space and pupil size for each eye. The data for both eyes were averaged, but data from a single eye were used when only one eye was detected. Eye data were recorded as null when no eye was detected. From the coordinates, we calculated the angles of horizontal gaze position using trigonometric function (Fig. 1a). All traces of gaze position, as well as pupil size, were previewed in MATLAB using a custom-designed graphic interface (Fig. 1b,c). The velocity of gaze position was obtained by differentiating the gaze position with respect to time. Initiation of a saccade was detected using the criterion that three consecutive velocity values were above 30°/s (Fig. 1c). It should be noted that this head-unrestrained eyetracking system measures saccadic gaze shift, which includes eye movements in the head plus head movement in space (i.e., changes of the visual axis in space). Monitoring of gaze shifts may be advantageous because the activity of saccade-related neurons in the superior colliculus encode gaze shift, rather than eye movements *per se*^26,27^. Saccadic RT was measured as the temporal interval between the onset of the target and the initiation of the saccade. Saccade data were included in the analysis when the RTs were between 0 and 500 ms. Pupil dilation was analysed as an indicator of saccadic motor preparation^28,29^ for expected target appearance. Because pupil size often exhibited a continuous decreasing-increasing pattern without any clear floor within a short inter-trial interval of 1,500 ms (Fig. 1b,c), a definite time point for a baseline could not be determined. Thus, the pupil size baseline was defined as the minimum value within the time window between 1000 ms and 0 ms before the target onset, during which gaze was fixated on the target. Pupil dilation was then defined as the change of the pupil size from the baseline to the target onset for each trial.

#### Extraction of reorienting process

When executing a saccade in response to an unexpected stimulus, people reorient their attention to it and thereafter generate a motor command to move the eyes. To extract the reorienting process from the task more precisely, we made the following assumptions. In the first jump, we assumed that participants had no information about the position and the timing of the target. Thus, the appearance of the target would be unexpected, requiring considerable time for reorienting. Within several subsequent jumps, however, participants could learn the sequence, meaning that the appearance of the target would become expected, so reorientation would take less time. Thus, when the RT of the first saccade is subtracted by the RT of the following saccades, the time required for the motor command is cancelled out, and the time for the reorienting process is extracted. Further, the data of the subsequent saccades were limited to odd trials (trial 3, 5, 7, 9), which had the same direction as the first saccade, because motor commands for ocular movement might be individually lateralised towards the right or left^30^. Data from the subsequent odd trials were averaged and named “subsequent trials”. Subtracted RTs of the first-minus-subsequent trial were then calculated. In short, subtracted RTs in a hemi-space may represent reorienting time in the contralateral hemisphere.

### Statistical analyses

To examine whether properties of the following saccades were differentiated from those of the first saccade, RTs were assessed using a mixed analysis of variance (ANOVA) with task (rightward vs leftward) as a between-subjects factor, and trial (trial 1, 3, 5, 7, 9) as a within-subjects factor. For this statistical analysis, missing RT values were replaced by the mean values of the previous and next trials. To evaluate the relationship between this task and the line bisection test, we conducted correlation analysis between the bisection performance and subtracted RTs for the rightward and leftward tasks. Statistical analyses were performed using SPSS version 23 (IBM, Japan).

## Results

### Line bisection performance

Data from 13 participants were excluded from the analysis because of detection failure of the first saccade (n = 11, one exhibited an RT of 516 ms for the first saccade) or any following odd saccades (n = 1, the subject had RTs below 0 ms in all of the following saccades) and unsaved data (n = 1). Additionally, two participants were removed because their eyes were positioned too far from the screen because of the lack of head restraint, producing smaller visual angles below 25° between the two target positions. We then analysed the remaining 39 participants (19 and 20 for the leftward and rightward tasks, respectively), who were all right-handed (mean [SD]: +92 [12]). Consistent with previous studies^8^, line bisection was deviated slightly toward the left at the group level for the leftward task group (−1.51 ± 2.36 mm; *t*_18_ = −2.79, *r* = 0.55, *p* = 0.012) and the rightward task group (−1.70 ± 2.13 mm; *t*_19_ = −3.55, *r* = 0.63, *p* = 0.002). There were no significant differences in age, handedness or line bisection deviation between the leftward and rightward tasks (Table 1).

**Table 1:** Demographics, RT and pupil dilation for analysed participants.

	Leftward task (n = 19)	Rightward task (n = 20)	*t*-value (df = 37)	Effect size (*r*)	*p*-value
Age [years]	36.0 (6.3)	37.5 (8.9)	−0.61	0.01	0.546
Handedness	92 (11)	92 (12)	−0.01	0.00	0.991
Line bisection deviation [mm]	−1.51 (2.36)	−1.70 (2.13)	0.26	0.04	0.779
**RT [ms]**					
First	328.8 (69.4)	304.0 (53.2)	1.26	0.20	0.216
Subsequent	227.4 (33.8)	211.6 (38.9)	1.35	0.22	0.184
Subtraction	101.5 (50.6)	92.4 (54.3)	0.54	0.09	0.596
**Pupil dilation [mm]**					
First	0.079 (0.078)^#^	0.079 (0.063)	0.00^$^	0.00	0.999
Subsequent	0.131 (0.076)^#^	0.166 (0.091)	−1.28^$^	0.21	0.207

Mean (S.D.), ^#^n = 17 for detection failure, ^$^df = 35.

### Differential properties of the following saccade from the first saccade

Because the head was not fixed by a head or chin rest, the distances between the eyes and the screen were variable, and therefore the visual angles of the target jumps varied across 38 participants (30.1 ± 2.2°). However, the jump angles were not significantly different between the first and subsequent trials (30.3° vs 30.1°; *t*_38_ = 1.52, *r* = 0.24, *p* = 0.138, paired *t*-test), and between the leftward and rightward tasks (29.4 vs 30.8°; *t*_37_ = −2.00, *r* = 0.31, *p* = 0.053, *t*-test).

**Figure 2:**
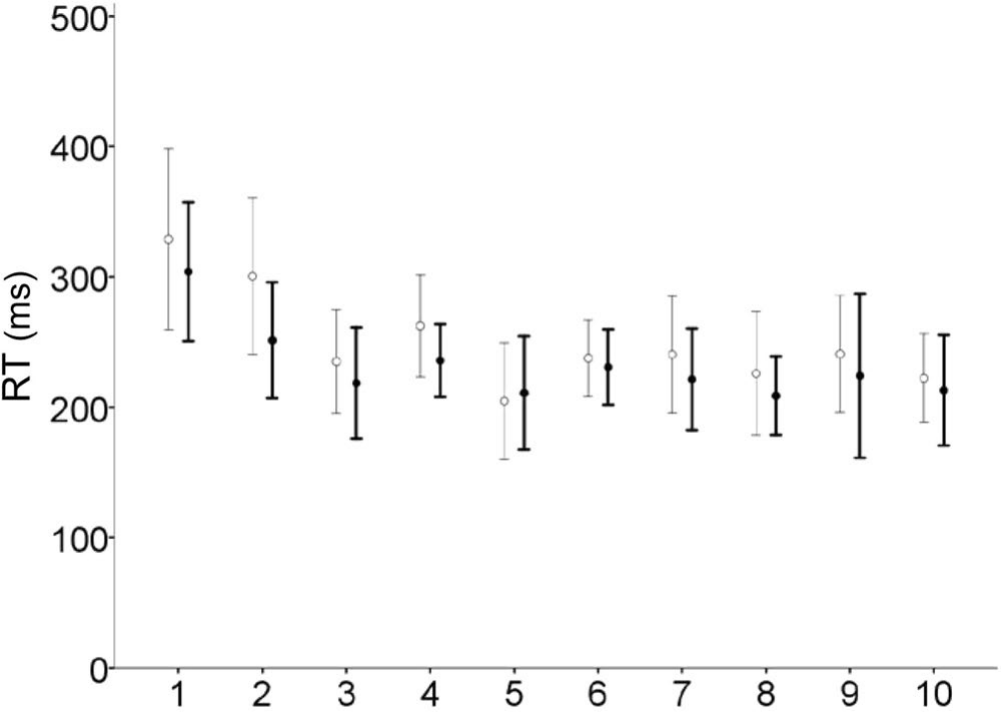
RTs of all trials. Thin and thick lines represent RTs in leftward and rightward task groups, respectively. The error bars represent ± SD across participants.

**Figure 3:**
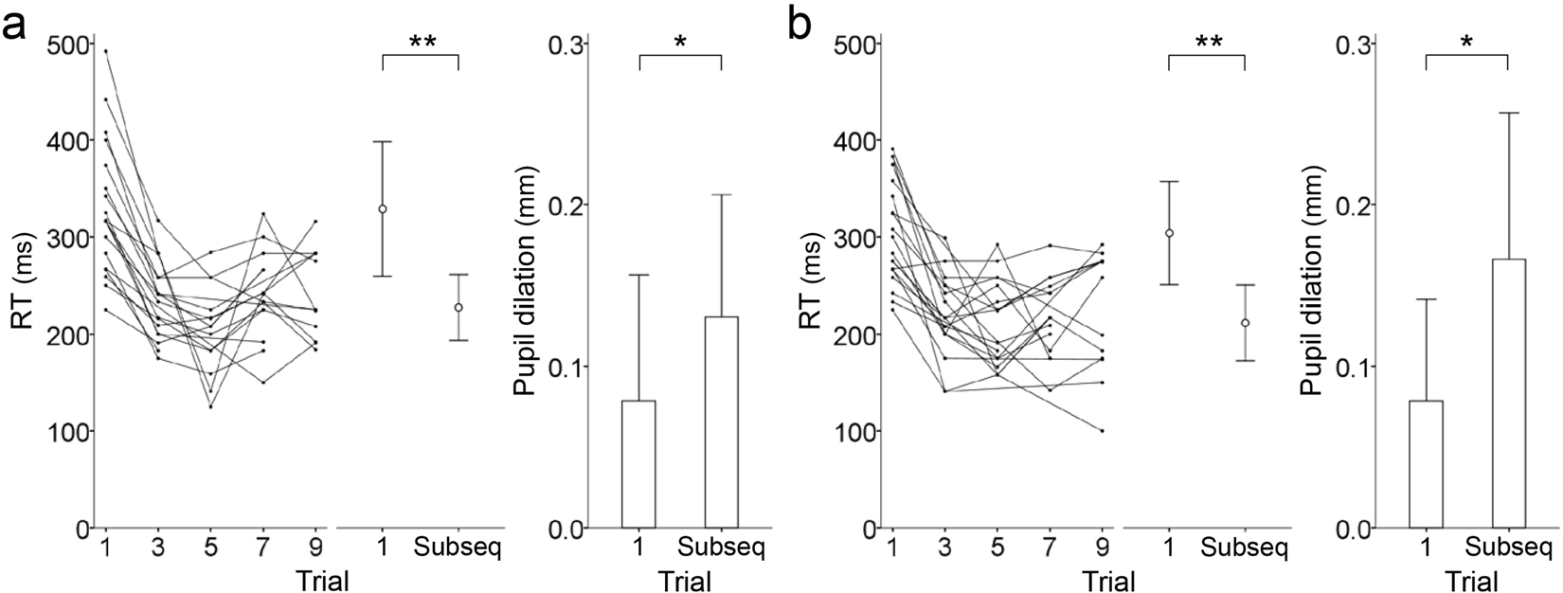
RTs of odd trials for leftward (**a**) and rightward tasks (**b**). Line charts (left) represent RT changes for each participant. The bar (middle) and column (right) charts show the mean RTs and pupil dilations, respectively, in the first and subsequent trials. The error bars represent ± SD across participants. “Subseq” means subsequent, that is, averaged data across the following trials (trial 3, 5, 7 and 9). **p < 1 × 10^−7^, *p < 0.01.

RTs rapidly decreased and reached a plateau from trial 1 through 10, for both leftward and rightward tasks (Fig. 2). For each task, we analysed the data of odd trials (trial 1, 3, 5, 7 and 9), which had the same saccade direction (Figs 1c and 3). Among a total of 195 values of RT in five odd trials for 39 participants, 35 values (18%) were missing. In the ANOVA, 16 out of the 35 values were replaced. There was a significant main effect of trial on RT (*F*_4,92_ = 32.78, *η*_*p*_^2^ = 0.59, *p* = 5.59 × 10^−17^). There was no significant main effect of task (*F*_1,23_ = 1.90, *η*_*p*_^2^ = 0.08, *p* = 0.182) and no significant interaction between task and trial (*F*_4,92_ = 0.75, *η*_*p*_^2^ = 0.03, *p* = 0.558), indicating that participants exhibited a similar pattern of RT change in the two tasks. Further pairwise comparisons with Bonferroni correction revealed shorter RTs in all of the following trials compared with those in the first trial (adjusted *p* values < 5 × 10^−5^) and no significant differences between each pair of all following trials. This indicated that the properties of the following saccades were similar to one another, but were clearly differentiated from those of the first saccade.

RTs in the subsequent trial were shorter than those in the first trial for leftward (*t*_18_ = −8.73, *r* = 0.90, *p* = 6.87 × 10^−8^, paired *t*-test) and rightward tasks (*t*_19_ = −7.61, *r* = 0.87, *p* = 3.50 × 10^−7^; Table 1, Fig. 3). Meanwhile, pupil dilations in the subsequent trial were larger than those in the first trial for leftward (*t*_16_ = 2.94, *r* = 0.59, *p* = 0.00971) and rightward tasks (*t*_19_ = 3.51, *r* = 0.63, *p* = 0.00236, paired *t*-test). None of the RT or pupil data were significantly different between the two tasks. As shown in Fig. 2, participants had not completely learnt the pattern of target appearance by the second trial. Thus, we also compared the second trial versus the subsequent even trials (trials 4, 6, 8 and 10). In both directions, RTs in the subsequent even trials were still shorter than those in the second trial, whereas pupil dilations showed no differences (Supplementary Fig. S2 for details).

### Correlations between subtracted RT and line bisection performance

**Figure 4:**
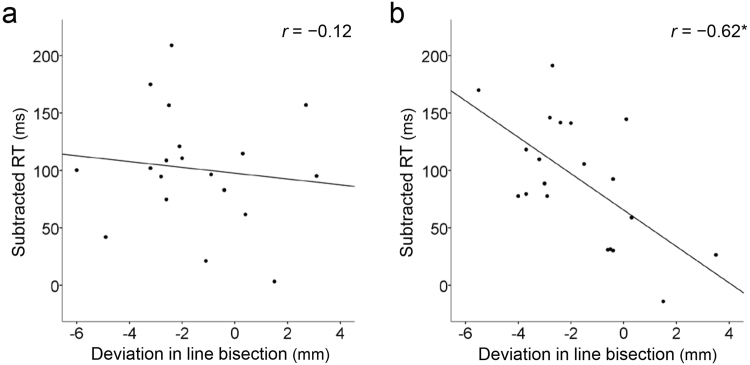
Correlations between line bisection deviation and subtracted RT for leftward (**a**) and rightward tasks (**b**) Linear regression lines are shown in the diagrams. *p < 0.05.

To examine the relationship between the saccade task and the line bisection test, we conducted a correlation analysis between bisection performance and the odd subtracted RT for leftward and rightward tasks (Fig. 4). For the rightward task, the results revealed a significant negative correlation, in which a larger left deviation in line bisection corresponded to a longer subtracted RT (*r* = −0.62, *p* = 0.003), while this correlation was absent in the leftward task (*r* = −0.12, *p* = 0.631). Conversely, the same analyses on the even trials revealed no correlations in either direction (Supplementary Fig. S3 for details).

## Discussion

Using a novel square wave saccade task, we investigated whether measuring saccades could detect lateralised visuospatial attention in neurologically intact adults. We found that subsequent saccades showed shorter RTs and greater pupil dilation than the first saccade. This finding indicates that the target was perceived as “unexpected” in the first saccade but became “expected” in the subsequent saccades and the subtracted RT of the first-minus-subsequent saccades could represent the time required for the reorienting process. The current findings also revealed that a longer subtracted RT for rightward saccades corresponded to greater deviation towards the left in the line bisection test. Because this correlation was absent in leftward saccades, the correlation between the subtracted RT and line bisection performance appears to be a hemispheric-specific finding.

The present results revealed no asymmetry in the subtracted RT between the rightward and leftward task groups. This result is seemingly contradictory to the group-level leftward bisection bias for the two task groups because the leftward bias indicates the superiority of the right hemisphere in visuospatial attention. However, the result is in accord with the findings of a seminal study by Thiebaut de Schotten *et al*.^6^. This previous study examined how quickly subjects detected a target that appeared in either the left or right hemifield and investigated the correlation between the lateralisation of RT and line bisection deviation. The researchers demonstrated that faster RTs in the left compared with the right hemifield corresponded to greater deviation toward the left in the bisection test (Fig. 2d in their paper). As shown in the figure, the bisection was significantly left-biased at the group level (−1.53 mm on average; 14 subjects exhibited leftward deviation and five exhibited rightward deviation) whereas RTs showed no asymmetry (eight subjects exhibited faster RTs in the left hemifield and 11 exhibited faster RTs in the right hemifield). Overall, it is possible that biological mechanisms are involved in correcting the asymmetries of target detection as a population, while individual variability could have remained present after this correction process, and was able to be detected using the present task paradigm.

The correspondence between greater left bisection deviation and longer subtracted RTs for rightward saccades may reflect individual abilities of the left hemisphere in visuospatial function. Greater left deviation has been interpreted as an overestimation of the left portion of the line compared with the right portion and can be attributed to greater involvement of the right than the left hemisphere^10,11^. In light of the relationship between the right and left hemispheres^31,32^, these findings could alternatively be interpreted as an underestimation of the right portion of the line and attributable to a lesser engagement of the left hemisphere. In contrast, the longer subtracted RT for rightward saccades suggests a slower reorienting process in the right hemi-space, indicating slower processing in the left hemisphere. Thus, if left hemisphere ability is inferior, participants would be expected to reorient their attention to a right-sided target slowly, and would tend to underestimate the right portion of a line, bisecting it towards the left.

The lack of the correlation for leftward saccades can be accounted for by a classical model in which the right hemisphere can shift attention to any region of space, whereas the left hemisphere only controls attention to the right side^1,3^. The model provides an explanation for the phenomenon of neglect, in which right hemispheric damage impairs attention to the left hemi-space while left hemispheric damage can be largely compensated. When the line bisection test is interpreted in terms of the model, the right hemisphere is thought to estimate both sides of a line, whereas the left hemisphere is involved in estimating the right side only. Therefore, line bisection performance may depend more directly on left than right hemisphere function. This may explain the lack of correlation in the leftward task in the current study.

Our separate analyses of each hemi-space provided a measure of the distinct characteristics of left hemisphere function in visuospatial processing. To assess hemispheric lateralisation, most previous studies have compared behaviours, functions and structures of the brain between the left and right hemi-spaces and/or hemispheres^6,21,33–36^. For example, Thiebaut de Schotten *et al*. demonstrated that individual asymmetry of line bisection performance and target detection time can be predicted by the lateralised volume of the superior longitudinal fasciculus II^6^. They found that subjects with smaller fasciculus volumes in the left hemisphere exhibited longer detection times in the right hemi-field, indicating slower processing speed in the left hemisphere. Whereas this previous study revealed individual variability in the processing speed of the left relative to the right hemisphere, the current study demonstrated variability in the left hemisphere *per se*.

The present square wave task might be regarded as a very short version of other types of cued response time paradigm, such as Posner’s task^37^ and the lateralised attentional network test^35^. In these paradigms, subjects are asked to detect a visual target presented in either the left or right visual field and make a manual response. The target is preceded by a cue that is likely (valid condition) or unlikely (invalid condition) to indicate the position at which the target will emerge. The appearance of a target is expected in the valid condition, leading to shorter RTs. Conversely, target appearance is unexpected in the invalid condition, resulting in longer RTs. Therefore, the targets in the invalid and valid condition have properties similar to those in the unexpected first and expected subsequent trials in the current study, respectively. Moreover, RTs in the valid condition subtracted from those in the invalid condition are considered to reflect reorienting processes^21,33^, like the subtracted RT in our study.

Pupil dilation could be a critical indicator of expectation regarding an upcoming stimulus. As shown in Fig. 1c, pupil size began to increase before the onset of the next target in subsequent saccades. A saccade is generally considered to be a specific kind of motor response^28^. Several studies have shown that pupil dilation begins before the time of a manual response or a saccade and is linked to the preparation of motor responses^28,29,38,39^. One previous study reported an association between greater pupil dilation and shorter RTs in pro-saccade tasks^28^. The current results suggested that smaller pupil dilation in the first trial may reflect unexpectedness of the target, related to longer RTs and evidence of the involvement of the reorienting process. This finding was supported by the analysis of the even trials, in which the bisection-subtracted RT correlation was not observed when longer RTs were not associated with smaller pupil dilation. Monitoring of pupil dilation may be valuable for verifying whether the task elicited the reorienting process, potentially elucidating the asymmetry of RT for visuospatial attentional function.

The current study involved several limitations that should be considered. First, the number of trials was relatively limited, particularly because we relied on the single first trial. In contrast, previous studies have almost always used averaged data from multiple repeated trials. This small trial number may have compromised the reliability of the data, and did result in the exclusion of 11 participants (20%) whose first saccade was not detected. However, the target at the first trial in the present paradigm was completely unexpected and therefore elicited the reorienting process more effectively than repeated targets. Although the data of the first trial are particularly important, previous studies have generally not differentiated it from data on subsequent trials, or have even removed first trial data from analyses as an outlier^23,24,40^. Next, the present paradigm measured the reorienting function only of either the right or left hemisphere, not both hemispheres, for each participant, meaning that the interaction between the two hemispheres could not be tested. To examine the interaction in future studies, we plan to develop a modified paradigm that keeps the horizontal location constant but varies the vertical location of the target, creating unexpectedness in both directions. Finally, the accuracies of the measured gaze position and pupil size may have been compromised by the calculation methodology^41,42^ and the lack of head restraint^20^. Previous studies have reported that gaze position can be affected by pupil size^41,42^ and vice versa^20^ in infrared eyetrackers. However, we measured pupil size at the same position of the target only in odd trials within each participant, and gaze position was not influenced by pupil size with visual inspection (Fig. 1c) for any participants. Further, the accuracy of gaze position was not crucial in our analyses because only the initiation times of saccades were needed for calculation of RT.

In conclusion, we demonstrated that the rightward saccades in the square wave paradigm can be used as a marker for lateralised visuospatial attention. The present task can be used as the basis for future research to further elucidate when and how lateralisation emerges and develops during childhood.

## Electronic supplementary material

Supplementary Information
