# Supplementary material for: A rightward saccade to an unexpected stimulus as a marker for lateralised visuospatial attention
Source: Sci Rep. 2018 May 15;8:7562. doi: 10.1038/s41598-018-25890-y (PMC5954050; doi:10.1038/s41598-018-25890-y)
Supplement: Supplementary file 1 — Supplementary Information [file 41598_2018_25890_MOESM1_ESM.pdf]

Supplementary information for

A rightward saccade to an unexpected stimulus as a marker for lateralised visuospatial attention

Masafumi Sanefuji, Hiroshi Yamashita, Michiko Torio, Daisuke Katsuki, Satoshi Akamine, Yoshito Ishizaki, Junji Kishimoto, Yasunari Sakai, Hidetoshi Takada, Keiko Yoshida and Shouichi Ohga

Supplementary Figure S1. Configuration of the experimental apparatus.

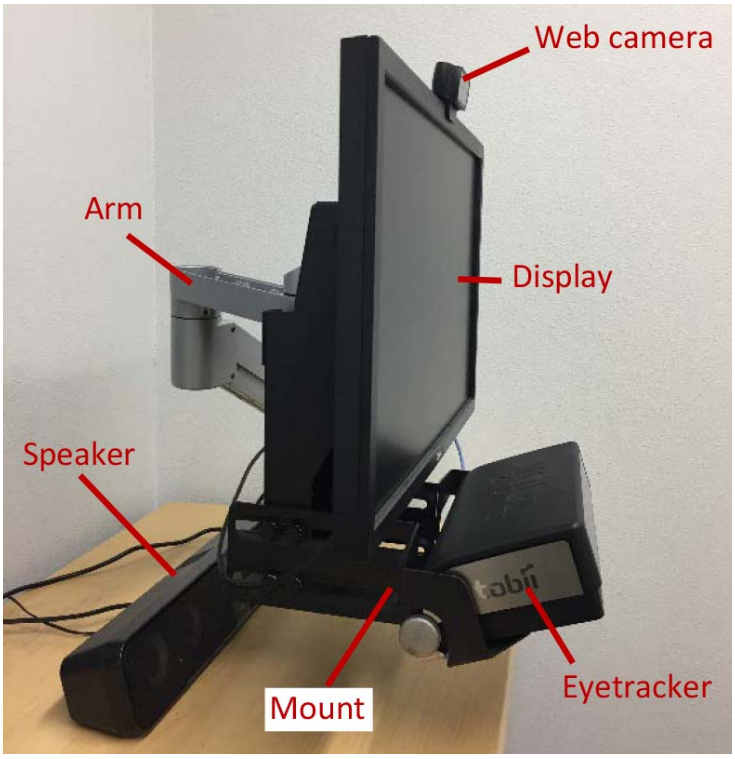

Permission to use the image that includes the Tobii logo was obtained from ©2018 Tobii AB.

Supplementary Figure S2. RTs of even trials for leftward saccades in the rightward task **(a)** and rightward saccades in the leftward task **(b)**.

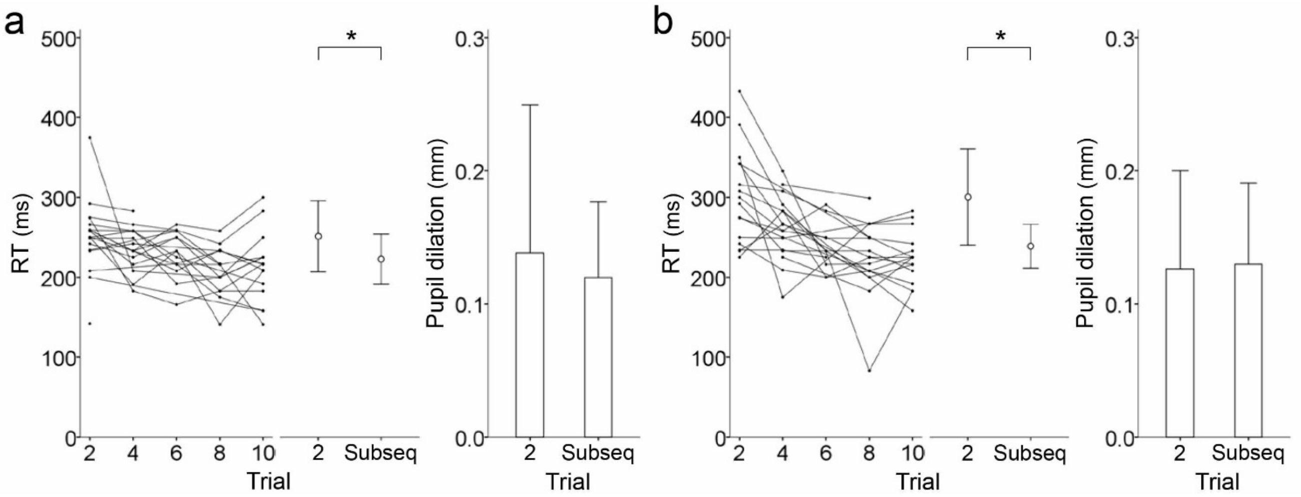

Line charts (left) represent RT changes for each participant. The bar (middle) and column (right) charts show the mean RTs and pupil dilations, respectively, in the second and subsequent even trials. The error bars represent ± SD across participants. "Subseq" means subsequent, that is, averaged data across the following even trials (trial 4, 6, 8 and 10). *$p < 0.01$. RTs in the subsequent even trial were shorter than those in the second trial for leftward saccades in the rightward task (222.9 ± 32.2 ms vs 257.4 ± 36.8 ms; $t_{17} = -3.47$, $r = 0.64$, $p = 2.94 \times 10^{-3}$, paired *t*-test) and for rightward saccades in the leftward task (236.3 ± 23.9 ms vs 300.4 ± 60.0 ms; $t_{15} = -4.08$, $r = 0.73$, $p = 9.85 \times 10^{-4}$, paired *t*-test). Conversely, pupil dilation showed no differences between the subsequent and the second trials for the leftward saccades (0.119 ± 0.057 mm vs 0.141 ± 0.114 mm; $t_{16} = -0.81$, $r = 0.20$, $p = 0.431$) and for the rightward saccades (0.130 ± 0.061 mm vs 0.126 ± 0.073 mm; $t_{14} = 0.14$, $r = 0.04$, $p = 0.887$).

Supplementary Figure S3. Correlations between line bisection deviation and subtracted RTs for leftward saccades in the rightward saccade task **(a)** and rightward saccades in the leftward task **(b)** in the analyses of even trials.

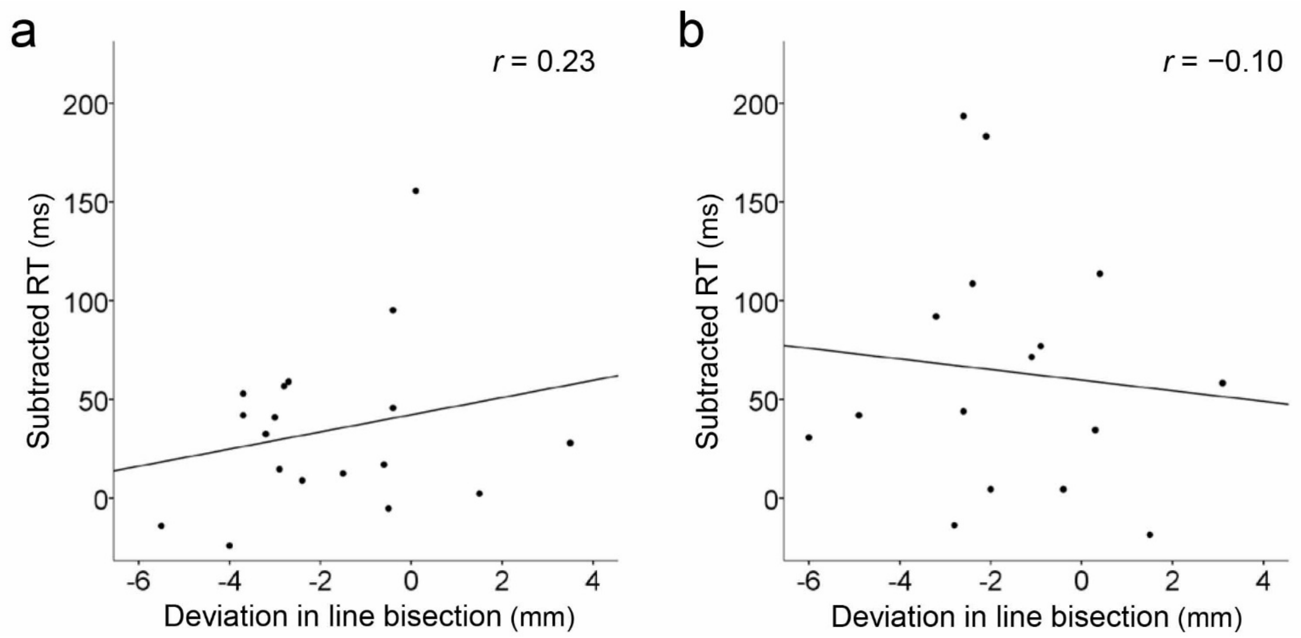

Linear regression lines are shown in the diagrams. There were no significant correlations for leftward saccades ($r = 0.23$, $p = 0.366$) or rightward saccades ($r = -0.10$, $p = 0.719$).
